# Justifying the principle of indifference

**DOI:** 10.1007/s13194-018-0201-0

**Published:** 2018-03-13

**Authors:** Jon Williamson

**Affiliations:** Philosophy Department, SECL, University of Kent, Kent, CT2 7NF UK

**Keywords:** Principle of indifference, Bayesianism, Epistemic consequentialism, Accuracy

## Abstract

This paper presents a new argument for the Principle of Indifference. This argument can be thought of in two ways: as a pragmatic argument, justifying the principle as needing to hold if one is to minimise worst-case expected loss, or as an epistemic argument, justifying the principle as needing to hold in order to minimise worst-case expected inaccuracy. The question arises as to which interpretation is preferable. I show that the epistemic argument contradicts Evidentialism and suggest that the relative plausibility of Evidentialism provides grounds to prefer the pragmatic interpretation. If this is right, it extends to a general preference for pragmatic arguments for the Principle of Indifference, and also to a general preference for pragmatic arguments for other norms of Bayesian epistemology.

Many Bayesians are committed to some version or other of the Principle of Indifference, which holds that in certain situations one’s degrees of belief should be equivocal. Section 1 introduces three such versions in order of increasing strength. In Section 2, I develop a consequentialist argument for the strongest version. This can be thought of as motivating the principle in terms of its *pragmatic* consequences: if one is to minimise worst-case expected loss, then one should satisfy the Principle of Indifference. As I explain in Section 3, an analogous argument can be constructed to motivate the strongest version of the principle in terms of its *epistemic* consequences: if one is to minimise worst-case expected epistemic inaccuracy, then one should satisfy the Principle of Indifference. In Section 4, we shall see that this sort of epistemic consequentialist argument conflicts with Evidentialism, which holds that one’s beliefs are epistemically rational if and only if they are compatible with one’s evidence. In Section 5 I argue that this is a worry not only for the epistemic justification presented in Section 3, but also for any epistemic justification of the Principle of Indifference: one should not be able to provide an argument for a Principle of Indifference purely in terms of its epistemic consequences, because the Principle of Indifference goes well beyond the evidence, and epistemic considerations should at most motivate conforming to the evidence. I argue that this concern also calls into question epistemic arguments for other Bayesian norms, such as Probabilism (the view that the strengths of one’s beliefs should be probabilities). For this reason, Bayesians are on safer ground motivating norms in terms of their pragmatic consequences, rather than their epistemic consequences.

## Three versions of the principle of indifference

Whilst radical subjectivist Bayesians would want to maintain that there are very few constraints on rational belief and would reject the Principle of Indifference (e.g., de Finetti 1937), many Bayesians are committed to some version or other of the Principle of Indifference. Objective Bayesians often explicitly endorse norms on belief which imply the Principle of Indifference, such as the Maximum Entropy Principle (Jaynes 1957; Williamson 2010). Moreover, as Hawthorne et al. (2017) argue, Bayesians who endorse any principle which requires calibration of degrees of belief to some probabilities of which one has evidence—e.g., the Principal Principle, the Reflection Principle, or any testimony principle which posits deference to expert authorities—are also committed to a version of the Principle of Indifference.

The Principle of Indifference has been formulated in many ways. In this paper we shall consider three versions, which will be introduced in this section in order of increasing strength.

We shall assume the following context in this section. First, we shall focus on a particular agent and suppose that there is a most fine-grained set Ω of mutually exclusive and exhaustive propositions that this agent can entertain or express: these are the agent’s *basic possibilities*. Any other expressible proposition can be thought of as a subset of Ω, the subset of possibilities in which that proposition is true. For example, a simple artificial agent may be speculating about the results of an experiment with three possible outcomes, *ω*_1_,*ω*_2_,*ω*_3_ respectively, in which case we might have Ω = {*ω*_1_,*ω*_2_,*ω*_3_}; if *ω*_1_ is a positive outcome then the proposition that outcome is not positive can be thought of as the subset {*ω*_2_,*ω*_3_}⊆Ω. Expressible propositions can thus be represented by members of the power set Ω of Ω. The Principle of Indifference is well known to lead to complications or inconsistencies on infinite domains, and its application there is very controversial (see, e.g., Keynes 1921, Chapter 4). For this reason, we restrict our attention here to the case in which Ω is finite. Having said that, it is worth pointing out that the principles of indifference which we discuss here can be extended in a consistent way to the case in which an agent’s language can be modelled as a first-order predicate language and the (infinitely many) basic possibilities represent truth assignments to the atomic propositions of the language—see Williamson (2017). To keep things simple, we shall suppose that the agent in question cannot express higher-order probability propositions—propositions *about* chances or *about* degrees of belief—if she could, we would have to consider a variety of further norms on degree of belief and specify how they interact with the Principle of Indifference.

Suppose that available evidence *E* constrains the agent’s belief function *P*, which represents her degrees of belief in the various propositions that she can express, to lie in some convex set 𝔼 of probability functions.^1^ For instance, evidence *E* might consist of a set of expressible propositions; in this case, *E* constrains belief function *P* to lie in the convex subset $\mathbb {E}$ of probability functions which give probability 1 to each proposition in *E*. To return to our simple example, if *E* contains only the proposition that the experiment did not yield a positive outcome, {*ω*_2_,*ω*_3_}, then 𝔼 = {*P* : *P*(*ω*_2_) + *P*(*ω*_3_) = 1} and 𝔼 is convex. In general, however, we shall not assume that the available evidence *E* consists entirely of propositions that the agent can express, i.e., propositions in the domain Ω of the belief function *P*. For example, evidence *E* might alternatively consist of evidence of chances, which, we have assumed, is not expressible in the sense outlined above. If *E* determines just that the chance function *P*^∗^ lies in convex 𝔼, a Bayesian who endorses some form of calibration to chances will want to say that the agent’s belief function *P* should also lie in $\mathbb {E}$. Thus if *E* says that *P*^∗^({*ω*_2_,*ω*_3_}) = 0.9, so *P*^∗^∈ 𝔼 = {*P* : *P*(*ω*_2_) + *P*(*ω*_3_) = 0.9}, then arguably also *P* ∈ 𝔼, which is a convex set of probability functions. If *E* is inconsistent, we shall take 𝔼 = *ℙ*, the whole space of probability functions on Ω.

**Fig. 1:**
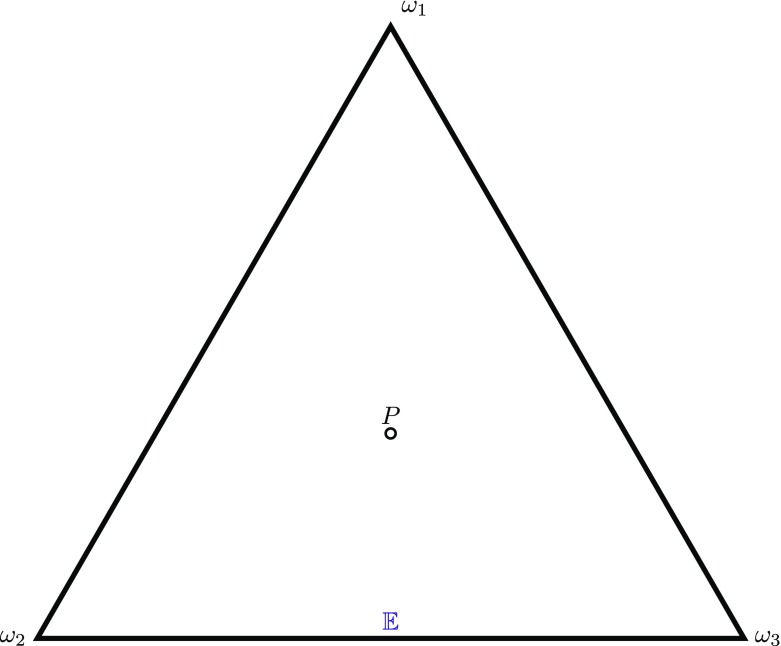
Visualisation of PI1

Let us now turn to the first of the three versions of the Principle of Indifference that we shall consider in this paper. This first version says that if there is no evidence at all then one should believe each basic possibility to the same extent:

- **PI1**: If *E* = *∅* then *P*(*ω*) = 1|Ω|, for each *ω* ∈Ω.
  We shall call the probability function that gives the same probability to each basic possibility the *equivocator function* and denote it by *P*_=_:
  $$P_=(\omega ) \stackrel{\text{df}}{=} \frac{1}{|{\Omega} |},\text{ for each}\,\, \omega \in {\Omega} .$$
  PI1 is visualised in Fig. 1. Here there are three basic possibilities, Ω = {*ω*_1_,*ω*_2_,*ω*_3_}. The triangle and its interior represent the set of probability functions: each vertex represents the function that gives probability 1 to the corresponding basic possibility and probability 0 to each of the other two; an edge represents the set of probability functions that give probability 0 to the basic possibility at the opposite vertex; all other probability functions are in the interior. If there is no evidence, *E* = *∅*, then 𝔼 is the entire set of probability functions on Ω, and PI1 says that *P* should be set to the equivocator function, the mid-point of the triangle, which gives probability 13 to each basic possibility.

The second version of the Principle of Indifference that we shall consider says that if the evidence treats each basic possibility symmetrically, then one should believe each such possibility to the same extent:

- **PI2**: If *E* is invariant under permutations of the *ω* ∈Ω, then *P* = *P*_=_.^2^

**Fig. 2:**
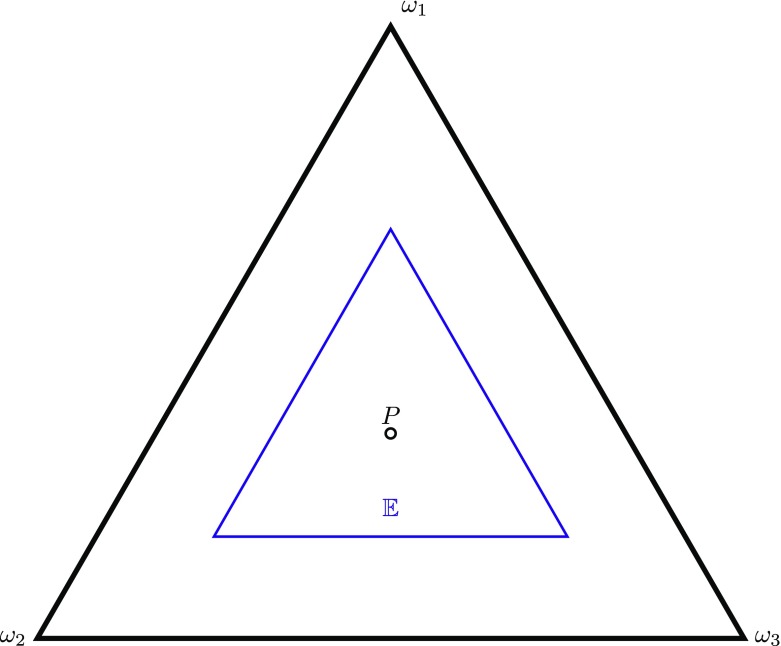
Visualisation of PI2

This implies PI1, since if *E* is empty then it is invariant under permutations of the basic possibilities. PI2 is depicted in Fig. 2. Here 𝔼 is a strict subset of the set of probability functions, invariant under 120^∘^ rotations.

The third version of the Principle of Indifference says that if it is compatible with the evidence to believe each basic possibility to the same extent, then one should do so:

- **PI3**: If *P*_=_ ∈ 𝔼 then *P* = *P*_=_.

**Fig. 3:**
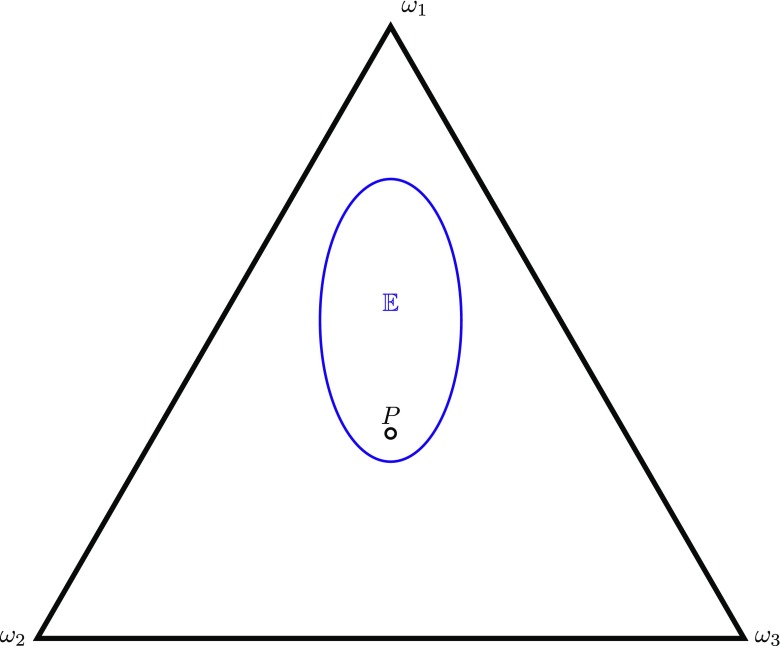
Visualisation of PI3

This principle, depicted in Fig. 3 is stronger still, implying PI2 and thereby also PI1.^3^

## Pragmatic justification: controlling loss

Having introduced some principles of indifference, in this section I shall develop a consequentialist argument for PI3 which appeals to some technical results of Landes and Williamson (2013). In this section, PI3 will be explicitly motivated in terms of its *pragmatic* consequences: it turns out that satisfying PI3 is advantageous in that it minimises worst-case expected loss. Later, in Section 3, we shall see that an argument with the same formal structure can be recast in *epistemic* consequentialist terms.

The argument presented here does more than justify the Principle of Indifference; it also justifies other Bayesian norms. In particular, it justifies Probabilism—the claim that *P* should be a probability function—and a calibration norm, which says that *P* should be calibrated to chances. Thus in this section, we shall not presume Probabilism, nor shall we assume that *P* should be calibrated to chances, as we did in Section 1. Instead, we shall derive these norms. Let $\mathbb {E}$ be the set of *evidentially-compatible chance functions*: evidence determines that the chance function *P*^∗^ lies within set 𝔼 ⊆ *ℙ*, and 𝔼 is the smallest such set (there is no strict subset 𝔼^′^⊂ 𝔼 such that the evidence determines that *P*^∗^∈ 𝔼^′^).^4^ I shall argue that the agent’s degrees of belief should be representable by a function *P* within the set $\mathbb {E}$. *P* is thus a probability function and is also calibrated to chances insofar as there is evidence of chances. In addition, we shall also see that *P* should satisfy PI3.

In this section we assume, purely for ease of exposition, that $\mathbb {E}$ is closed (i.e., contains its limit points) as well as convex. As before, we presuppose a finite set Ω of basic possibilities, e.g., Ω = {*ω*_1_,*ω*_2_,*ω*_3_}, and we construe expressible propositions as subsets of Ω, e.g., *F* = {*ω*_2_,*ω*_3_}. A *partitionπ* of propositions is a set of mutually exclusive and exhaustive subsets of Ω. For example, {{*ω*_1_},{*ω*_2_,*ω*_3_}} is a partition of propositions. π will denote the set of all partitions of propositions.

We shall suppose that an agent’s degrees of belief can be represented by a function *bel* : Ω→*ℝ*_≥ 0_ which attaches a non-negative real number to each expressible proposition. For example, one such belief function might set *bel*({*ω*_1_}) = 6,*bel*({*ω*_2_,*ω*_3_}) = 9,…. The set of belief functions is a much wider class of functions than the set of probability functions. I shall argue that, in order to minimise worst-case expected loss, the agent’s belief function should be a probability function, in $\mathbb {E}$, which satisfies the Principle of Indifference PI3.

### Normalisation

First, as a technical convenience, we shall normalise the belief functions. Roughly speaking, we divide all degrees of belief by the maximum amount of belief distributed amongst a partition of propositions. More precisely, for *M* = max*π*∈π∑ _*F*∈*π*_*bel*(*F*) we normalise belief function *bel* by considering instead belief function *B* : Ω→[0,1] defined by:

$$B(F) = \frac{\mathit{bel}(F)}{M}\text{ for all} F\subseteq {\Omega} .$$

For example, if *bel*({*ω*_1_}) = 6,*bel*({*ω*_2_,*ω*_3_}) = 9,*bel*({*ω*_2_}) = 12,*bel*({*ω*_3_}) = 12,… and the partition of propositions {{*ω*_1_},{*ω*_2_},{*ω*_3_}} is given maximum belief, *M* = 30, then *bel* would be normalised to give *B*({*ω*_1_}) = 0.2,*B*({*ω*_2_,*ω*_3_}) = 0.3,*B*({*ω*_2_}) = 0.4,*B*({*ω*_3_}) = 0.4,…. The symbol B will denote the set of all normalised belief functions. For each such function *B*,

$$\sum\limits_{F\in \pi} B(F)\! \leq \! 1\text{ for all}\,\, \pi \in {\Pi} ,\text{ and} \sum\limits_{F\in \pi} B(F)= 1\text{ for some}\,\, \pi \in {\Pi} ,$$

in virtue of the procedure used to normalise *B*. Note that the set $\mathbb P$ of all probability functions *P* : Ω→[0,1] is a subset of $\mathbb B$, since,

$$\sum\limits_{F\in \pi} P(F)= 1\text{ for all}\,\, \pi \in {\Pi} .$$

**Fig. 4:**
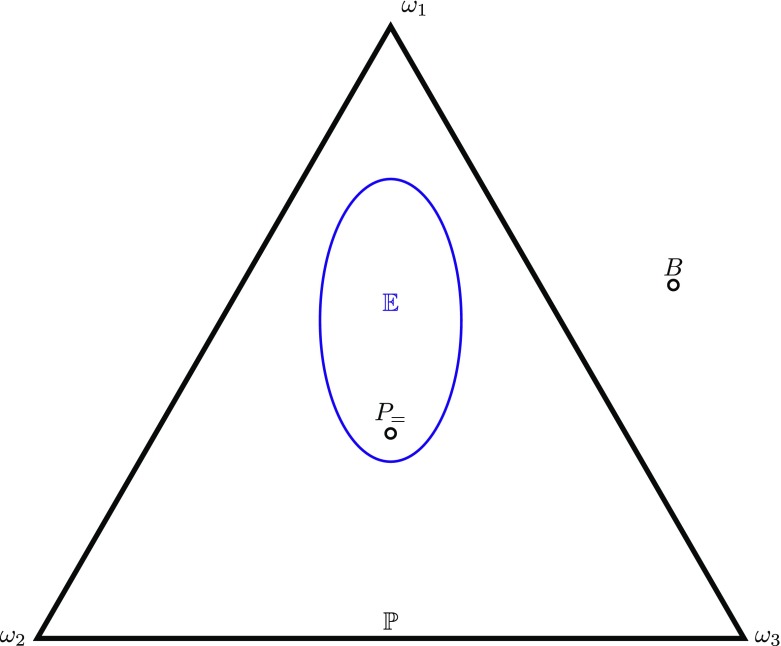
Belief function *B* outside the space $\mathbb P$ of probability functions

Thus if *B*({*ω*_1_}) = 0.2,*B*({*ω*_2_,*ω*_3_}) = 0.3 then *B* is not a probability function. The task is to show that whilst in general a belief function *B* may lie outside the space of probability functions (Fig. 4), in order to minimise worst-case expected loss the agent’s belief function should lie inside 𝔼 ⊆ *ℙ* and should be the equivocator function *P*_=_, if the equivocator function is in $\mathbb {E}$. This will establish PI3.

### Loss

Next, we need to be more specific about the notion of loss under consideration. Suppose the agent is not aware of the choices she will need to make, and is thus not aware of the actual losses (or gains) which will be incurred by her degrees of belief. What should she expect of her losses? Let *L*(*F*,*B*) denote the loss (aka disutility) one should anticipate will be incurred by adopting belief function *B* when *F* turns out to be true.^5^ We shall interpret this as the loss *specific to* *F*, i.e., the loss in isolation from losses incurred by *B* on other propositions which may be implied by *F* or which may imply *F*. We shall suppose:

- **L1**: One should not anticipate any loss when one fully believes a proposition that turns out to be true: *L*(*F*,*B*) = 0 if *B*(*F*) = 1. (Fully believing the truth leads to the best outcome.)
- **L2**: One should anticipate that the loss *L*(*F*,*B*) will strictly increase as *B*(*F*) decreases from 1 towards 0. (The less credence one gives to the truth, the worse the outcome.)
- **L3**: *L*(*F*,*B*) should depend only on *B*(*F*), not on *B*(*F*^′^) for *F*^′^≠*F*. (This represents the interpretation of *L*(*F*,*B*) as the loss incurred on *F* in isolation from that incurred on other propositions.)
- **L4**: Losses should be presumed to be additive when the space Ω of basic possibilities is generated by independent subspaces: whenever *ω* ∈Ω takes the form *ω*_1_ ∧ *ω*_2_ where *ω*_1_ ∈Ω_1_ and *ω*_2_ ∈Ω_2_, *B*(Ω) = 1, and Ω_1_ and Ω_2_ are independent in the sense that *B*(*F*_1_ × *F*_2_) = *B*(*F*_1_)*B*(*F*_2_) for all $F_{1}\times F_{2} = \{\omega _{1}\wedge \omega _{2} : \omega _{1}\in F_{1}, \omega _{2}\in F_{2}\}$, then $L(F_{1}\times F_{2},B) = L_{1}(F_{1}, B_{\downharpoonright {\Omega }_{1}}) + L_{2}(F_{2}, B_{\downharpoonright {\Omega }_{2}})$, where $L_{1}$ and $L_{2}$ are the loss functions on Ω_1_ and Ω_2_ respectively.

It turns out that these conditions force the loss function to be logarithmic:

$$L(F,B) = -k \log B(F),$$

for some positive constant *k* (Landes and Williamson 2013, Theorem 1).

### Expected loss

Next, let us consider expected loss. If the chance function is *P*^∗^, what is the expected loss incurred by *B* over all the expressible propositions? A function which measures expected loss is called a *scoring rule*.^6^ In fact, there are a range of plausible scoring rules. For example, both the following measures seem equally reasonable:

$$S_{{\Pi}} (P^{*},B) \stackrel{\text{df}}{=} \sum\limits_{\pi \in {\Pi}} \sum\limits_{F\in \pi} P^{*}(F) L(F,B),$$

$$S_{{\mathcal P}{\Omega}} (P^{*},B) \stackrel{\text{df}}{=} \sum\limits_{F\subseteq {\Omega}} P^{*}(F) L(F,B).$$

Since there is no single scoring rule that stands out as being most appropriate, we shall consider a whole class of scoring rules, indexed by a function *g* : π→*ℝ*_≥ 0_ which attaches a non-negative weight to each partition:

$$S_{g}(P^{*},B) \stackrel{\text{df}}{=} \sum\limits_{\pi \in {\Pi}} g(\pi ) \sum\limits_{F\in \pi} P^{*}(F) L(F,B).$$

*S*_π_ corresponds to the case in which each partition receives weight 1; *S*_Ω_ corresponds to the case in which each partition of size 2 receives weight 1 and all other partitions get weight 0.^7^

We shall impose two conditions on *g*. First, *g* is *inclusive*: every proposition *F* is in some partition that is given positive weight (otherwise, that proposition will not contribute to the score). Second, *g* is *unbiased*: it is invariant under permutations of the basic possibilities *ω* ∈Ω (otherwise, some possibilities are singled out *a priori* as more important than others). The weighting functions for *S*_π_ and *S*_Ω_ satisfy these two conditions.

Given the anticipated loss function set out above, we have that

$$S_{g}(P^{*},B) = -k \sum\limits_{\pi \in {\Pi}} g(\pi ) \sum\limits_{F\in \pi} P^{*}(F) \log B(F).$$

### Worst-case expected loss

Now, the precise chance function *P*^∗^ will usually not be known, so the expected loss is not fully accessible to the agent. The evidence only determines that *P*^∗^ lies in the set $\mathbb {E}$ of evidentially-compatible chance functions. The evidence does, however, permit us to calculate the worst-case expected loss,

$$\sup_{P^{*}\in \mathbb{E}} S_{g}(P^{*},B).$$

We can thus ask, which belief function *B* would incur minimum worst-case expected loss? I.e., which *B* achieves

$$\inf_{B\in \mathbb B} \sup_{P^{*}\in \mathbb{E}} S_{g}(P^{*},B)?$$

It turns out (see Landes and Williamson 2013, Theorem 2) that the belief function *B* ∈ B that incurs minimum worst-case expected loss is the probability function in $\mathbb {E}$ which has maximum *generalised entropy*:

$$H_{g}(P) \stackrel{\text{df}}{=} - \sum\limits_{\pi \in {\Pi}} g(\pi ) \sum\limits_{F\in \pi} P(F) \log P(F).$$

**Fig. 5:**
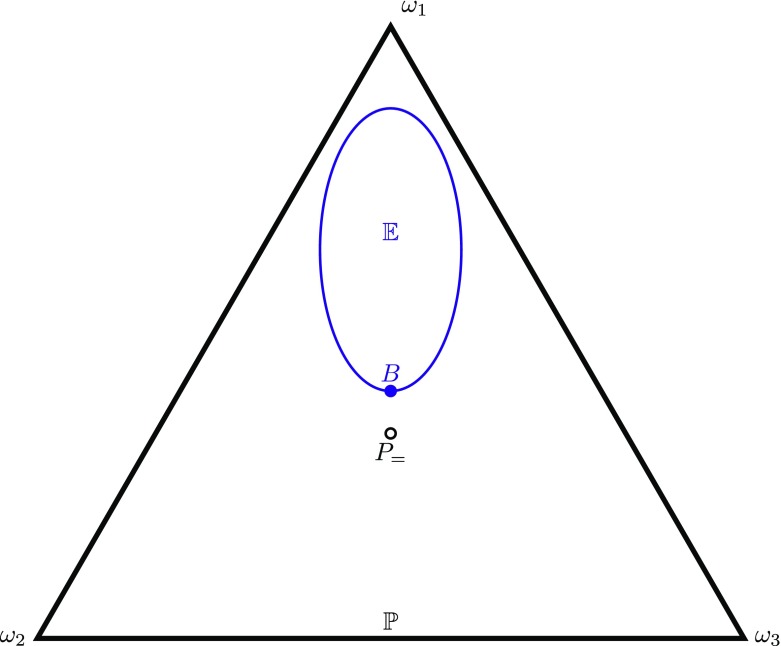
The optimum belief function B is the calibrated probability function with maximum generalised entropy

This is depicted in Fig. 5. Since *B* ∈ 𝔼 ⊆ *ℙ*, this establishes that the optimal belief function *B* is a probability function (i.e., Probabilism) and that the optimal belief function *B* is in the set $\mathbb {E}$ of evidentially-compatible chance functions (Calibration).

Moreover, the equivocator function is the probability function in *ℙ* that has maximum generalised entropy (Landes and Williamson 2013, Corollary 6). Hence, if the equivocator function is in $\mathbb {E}$ then it is bound to be the belief function that minimises worst-case expected loss. This establishes that if *P*_=_ ∈ 𝔼 then the optimal belief function *B* is the equivocator function *P*_=_. This is PI3, the strongest of the three principles of indifference. Note that PI3 still holds even if we drop the assumptions of closure and convexity of the set $\mathbb {E}$ of evidentially-compatible chance functions (Landes and Williamson 2013, Theorem 3).

## Epistemic justification: reducing inaccuracy

The above argument for the Principle of Indifference sought to justify it in terms of its pragmatic consequences: PI3 needs to hold if one is to minimise worst-case expected loss. Interestingly, exactly the same formal argument can be cast in terms of epistemic consequences. The only change involves interpreting the function *L* as a measure of epistemic inaccuracy, instead of a loss function. Thus our four desiderata become:

- **E1**: *L*(*F*,*B*) = 0 if *B*(*F*) = 1. (Fully believing the truth yields no inaccuracy.)
- **E2**: Inaccuracy *L*(*F*,*B*) strictly increases as *B*(*F*) decreases from 1 towards 0. (The less credence one gives to the truth, the greater the inaccuracy.)
- **E3**: Inaccuracy *L*(*F*,*B*) depends only on *B*(*F*), not on *B*(*F*^′^) for *F*^′^≠*F*. (This represents the interpretation of *L*(*F*,*B*) as the inaccuracy of one’s degree of belief in *F*, in isolation from inaccuracy on other propositions.)
- **E4**: Inaccuracy is additive when the space Ω of basic possibilities is generated by independent subspaces: whenever *ω* ∈Ω takes the form *ω*_1_ ∧ *ω*_2_ where *ω*_1_ ∈Ω_1_ and *ω*_2_ ∈Ω_2_, *B*(Ω) = 1, and Ω_1_ and Ω_2_ are independent in the sense that *P*^∗^(*F*_1_ × *F*_2_) = *P*^∗^(*F*_1_)*P*^∗^(*F*_2_) for all *F*_1_ × *F*_2_ = {*ω*_1_ ∧ *ω*_2_ : *ω*_1_ ∈ *F*_1_,*ω*_2_ ∈ *F*_2_}, then $L(F_{1}\times F_{2},B) = L_{1}(F_{1}, B_{\downharpoonright {\Omega }_{1}}) + L_{2}(F_{2}, B_{\downharpoonright {\Omega }_{2}})$, where $L_{1}$ and $L_{2}$ are the inaccuracy functions on Ω_1_ and Ω_2_ respectively.

The only formal difference here is that E4 appeals to objective independence, i.e., independence with respect to the chance function *P*^∗^, rather than subjective independence with respect to the belief function *B*.

Everything else follows through as before, but under an epistemic interpretation rather than a pragmatic interpretation. The four conditions set out above force the inaccuracy measure to be logarithmic:

$$L(F,B) = -k \log B(F).$$

A scoring rule now measures expected inaccuracy, rather than expected loss. As before, we consider consider a whole class of scoring rules generated by the inclusive and unbiased weighting functions. For any such weighting function, the (normalised) belief function which minimises worst-case expected inaccuracy is a probability function, in the set $\mathbb {E}$ of functions that are calibrated to chances, that has maximum generalised entropy. In particular, this optimal belief function satisfies PI3. The Principle of Indifference can thus be motivated in terms of epistemic rationality: supposing that epistemic rationality requires minimising worst-case expected inaccuracy, degrees of belief must then satisfy principle of indifference PI3.

We thus appear to be spoilt for choice when justifying the Principle of Indifference. The formal argument for the Principle of Indifference can be given either a pragmatic reading, which appeals to the notion of anticipated loss, or an epistemic reading, which appeals to the notion of inaccuracy. The question arises, should one prefer an epistemic justification over a pragmatic justification or vice versa?

Rather than evaluate the details of the particular argument presented above, I will suggest a general answer to this question, in the hope that a general answer will remain pertinent should new pragmatic or epistemic arguments for the Principle of Indifference be put forward in the future. The answer that I will present is that the pragmatic line of argument should be preferred, because of an inconsistency between the epistemic line of argument and Evidentialism.

## Evidentialism

In this section we shall explore the Evidentialism thesis and we shall see that this thesis is inconsistent with the epistemic line of argument presented above.

### Evidentialism

Consider the following principle: one’s beliefs are rational if and only if they are compatible with one’s evidence. There are compelling reasons to think that the ‘if’ is too strong here: rationality seems to demand more than compatibility with evidence. For example, suppose that a patient has evidence that the chance that she will survive her cancer (*s*) given a certain genetic profile (*g*) is 0.8, i.e., *P*^∗^(*s*|*g*) = 0.8, and that the chance she has genetic profile *g* is 0.7, i.e., *P*^∗^(*g*) = 0.7. Regarding survival, these two facts imply that *P*^∗^(*s*) ∈ [0.56,0.86].^8^ Assuming that one should calibrate degrees of belief to chances, insofar as one has evidence of them, the patient should believe *s* to some degree within the same interval, *P*(*s*) ∈ [0.56,0.86]. Now, it is known that a high degree of belief in survival after cancer influences the chance of survival itself (e.g., Soler-Vila et al. 2005). It is thus in the patient’s interest that she adopts a degree of belief in *s* that is at the higher end of the range. So there is a sense in which the patient’s degree of belief would be irrational were it not sufficiently close to 0.86: rationality requires more than mere compatibility with the evidence, because a degree of belief of 0.56 is compatible with the evidence yet apparently irrational. This sense of rationality is pragmatic however—it is motivated by the need to survive, rather than the quest for truth. There is nothing to say that a higher degree of belief is epistemically more appropriate than a lower degree of belief. After all, just as higher degrees of belief lead to higher chances of survival, so do lower degrees of belief lead to lower chances of survival—we may suppose here that all degrees of belief within the interval [0.56,0.86] are equally well calibrated to the chances.^9^

This sort of example, which shows that there can be pragmatic grounds for going beyond the evidence, suggests a reformulation of the above principle:

**Evidentialism.**: One’s beliefs are *epistemically* rational if and only if they are compatible with one’s evidence.
  The motivation behind this principle is that the evidence provides all we have to go on in our quest for truth. Advocates of Evidentialism include Conee and Feldman (2004) and McCain (2014).

Having stated the Evidentialism principle, let us consider some points of clarification.

When formulating evidentialism, ‘epistemically justified’ is sometimes used instead of ‘epistemically rational’. Thus Conee and Feldman (2004, p. 83) provide the following formulation of evidentialism: ‘Doxastic attitude *D* towards proposition *p* is epistemically justified for *S* at *t* if and only if having *D* towards *p* fits the evidence *S* has at *t*.’ The two terms are often used interchangeably, although ‘epistemically rational’ could be interpreted as a slightly more permissive classification than ‘epistemically justified’: that beliefs are justified suggests that there exists some justification that rules them in as appropriate beliefs, whereas beliefs might be classified as rational just in case there are no grounds for ruling them out as irrational. Here we shall stick with ‘epistemically rational’, which is more familiar in the Bayesian context. I use ‘one’s beliefs’ to denote a belief state—perhaps a set of propositions in the case of qualitative beliefs or a belief function in the case of Bayesian degrees of belief.

DeRose (2000, §1) notes that there is a sense in which one ought not believe a proposition unless one believes it *on the basis of* one’s evidence—i.e., one ought not believe it for mistaken reasons, even if that belief is, in fact, compatible with one’s evidence. As DeRose (2000) suggests, this consideration points to a distinction between different kinds of ‘oughts’: a strong notion which presumes correct ‘basing’ and the weaker notion of the Evidentialism thesis as stated above. The distinction can be put thus: one *rationally believes* if and only if one’s *beliefs are rational* (i.e., one’s belief state is compatible with one’s evidence) and that belief state is properly believed on the basis of one’s evidence. The stronger sense of rationality is clearly a different sense than that employed in the standard Bayesian notion of rational degree of belief. The Bayesian notion is oblivious to the actual genesis of beliefs, caring only whether the values in question are appropriate in the circumstances.

There is a further ambiguity that needs to be addressed. Baehr (2009) suggests that, whilst there is a sense of epistemic rationality in which Evidentialism is true, there is another sense in which the epistemic rationality of one’s beliefs depends on whether the gathering of evidence was defective. According to this stronger ‘ought’, one ought to gather enough evidence and gather it correctly, as well as ensure that beliefs are appropriate given that evidence. This is a rather complex ‘ought’: the individual who deliberately or negligently sees no evil and hears no evil may be both epistemically defective (as is someone who sees and hears evil but fails to believe it) and morally defective (as is someone who sees, hears and believes evil but fails to speak out against it). Again, there is no need for the Bayesian to deny this stronger ‘ought’—it suffices to observe that the Bayesian is primarily interested in the weaker sense of epistemic rationality. As in the case of believing on the basis of the evidence, it is plausible that an adequate account of the stronger ‘ought’ will need to invoke an adequate account of the weaker notion as a component, so Evidentialism will be at least part of the story.

It is important to note that Evidentialism as stated above is simply a biconditional claim, a characterisation of rational belief rather than an analysis of it. It is therefore important to distinguish Evidentialism from an evidentialist epistemology, i.e., a detailed epistemological theory under which Evidentialism turns out to be true. Such an epistemological theory would have as a minimum to give detailed accounts of: rationality; the distinction between epistemic and pragmatic rationality; doxastic deliberation; compatibility; the nature of evidence; and what it is to possess evidence. Work towards an evidentialist epistemology from an internalist point of view can be found in Conee and Feldman (2004), Dougherty (2011) and McCain (2014), for example, and Williamson (2000) develops an evidentialist epistemology from the externalist perspective (see Williamson 2000, §9.8). Clearly, one does not need to provide a detailed evidentialist epistemology in order to advocate Evidentialism.

### Bayesian Evidentialism

Whilst advocates of Evidentialism are not bound to provide a detailed theory of each of the terms that occur in the Evidentialism claim, it is incumbent upon proponents of the claim to clarify when it applies. In particular, it is important to be clear about what a belief state is and when a belief state is compatible with evidence. Given our concern with norms of Bayesian rational degree of belief in this paper, we shall explicate Evidentialism by appealing to the concepts set out in Sections 1 and 2. Thus a belief state is construed as a (normalised) belief function *B* and a belief function *B* is compatible with evidence just in case *B* ∈ E, the set of evidentially-compatible chance functions.

The motivation behind this explication of compatibility with the evidence appeals to the idea that degrees of belief should be calibrated to chances (a principle common to both the pragmatic approach and the epistemic approach under consideration here). Recall that 𝔼 is defined in Section 2 as the smallest set of probability functions that contains the chance function *P*^∗^, as far as can be determined by the evidence *E*. If beliefs should be calibrated to chances then a belief function is compatible with evidence just when it is calibrated to a chance function that is compatible with evidence. This yields the claim that *B* is compatible with the evidence if and only if *B* ∈ E. This claim can be motivated more precisely as follows.

First we shall see that if *B*∉E then *B* is incompatible with evidence. Suppose first that *E* is a set of expressible propositions—propositions that are not statements about chances. Then 𝔼 is the set of all probability functions that give maximal probability to propositions in *E*, 𝔼 = {*P* ∈ *ℙ* : *P*(*𝜃*) = 1 for all *𝜃* ∈ *E*}. Now if *B*∉E then there is some *𝜃* ∈ *E* such that *B*(*𝜃*) < 1. Such a belief function would be problematic. It would be Moore-paradoxical to recognise that *𝜃* is evidence yet not to fully believe *𝜃*. In general, one ought to calibrate one’s beliefs to truths, insofar as one has evidence of them, yet this belief function is not calibrated. Hence, this belief function *B* is incompatible with evidence. This argument can be extended to the case in which *E* provides evidence of non-trivial chances. If *B*∉E then there is some *𝜃* such that *B*(*𝜃*)≠*P*^∗^(*𝜃*) for any *P*^∗^∈ E. This is also problematic: one ought to calibrate one’s degrees of belief to chances, insofar as one has evidence of them, yet this belief function is not calibrated. Hence, this belief function is incompatible with the evidence.

On the other hand, if *B* ∈ 𝔼 then *B* is compatible with the evidence, for the following reason. That *B* ∈ 𝔼 means that as far as can be determined from *E*, *B* may well be the true chance function. Suppose new evidence is subsequently obtained that determines that *B* is indeed the true chance function *P*^∗^. This new evidence is clearly compatible with the old evidence *E*. Call the new evidence base *E*^′^ and the new set of evidentially-compatible chance functions E^′^. Now, *B* is compatible with *E*^′^: *E*^′^, we may assume, is consistent, so there must be some belief function compatible with *E*^′^; however, as we have just seen, any belief function outside $\mathbb {E}^{\prime }$ is incompatible with *E*^′^; hence, *B*, the only function in E^′^, is compatible with *E*^′^. Since *B* is compatible with *E*^′^ as a whole, it must be compatible with each item of evidence in *E*^′^. Hence it is compatible with each item of evidence in *E* ⊆ *E*^′^, and therefore with *E* as a whole.

This provides some motivation for the view that belief function *B* is compatible with evidence just when *B* ∈ E. Below, we will revisit the question whether this characterisation is appropriate. In the meantime, we are now in a position to state a Bayesian explication of Evidentialism:

- **BE** Belief function *B* is epistemically rational if and only if *B* ∈ E.

We shall call this specialisation of Evidentialism to degree of belief *Bayesian Evidentialism*.

Note that BE circumvents one immediate concern with Evidentialism in the Bayesian framework. This is the concern that Probabilism appears to conflict with Evidentialism. For example, according to Probabilism, one ought to fully believe all logical truths, even those for which one has no evidence that they are logical truths. Thus one ought to fully believe that the millionth digit of *π* is 5, even if one has no evidence that this is so. Probabilism, then, seems to be a constraint on rational degree of belief that operates independently of the evidence, contrary to Evidentialism. In response to this concern, it suffices to point out that Probabilism is treated as an idealisation by the Bayesian: Probabilism is usually advocated on the grounds that it is a simple and powerful approximation to a more nuanced, correct norm. In some cases, such as the application of Bayesianism to mathematics (Corfield 2001), one may need to invoke a more nuanced norm, but Probabilism suffices for most practical applications of Bayesianism. BE circumvents these issues by building Probabilism into compatibility with the evidence: since 𝔼 is construed as the set of chance functions compatible with the evidence, it is a subset of *ℙ*, the set of all probability functions. Thus there is no conflict between Probabilism and BE. This means that BE should also be treated as an idealisation—an approximation to a more nuanced claim that would incorporate the more nuanced, correct norm which Probabilism approximates.

Another point of clarification. In the cancer example at the start of this section, we supposed that all degrees of belief within the interval [0.56,0.86] were equally well calibrated to the chances. Consider a modification to the example where this is not the case: suppose that individuals with degree of belief *P*(*s*) ≥ 0.86 have chance 0.86 of survival, those with degree of belief *P*(*s*) ≤ 0.56 have chance 0.56 of survival and those with degree of belief strictly between 0.56 and 0.86 have some chance of survival *other than* that degree of belief, and that these facts are part of the patient’s evidence. In this case, only the endpoints of the interval, 0.86 and 0.56, are possible values of degrees of belief that are calibrated to the chances. This modified example shows that, in general, some chance functions that are compatible with evidence may not also be interpretable as belief functions compatible with evidence. The set $\mathbb {E}$ was construed in Section 2 as the set of chance functions that are compatible with the evidence. In its role in BE, $\mathbb {E}$ also needs to be construed as a set of belief functions compatible with the evidence. Thus we must take $\mathbb {E}$ to be the set of those chance functions compatible with evidence *to which belief functions can be calibrated*. This qualification avoids a worry about Evidentialism of Reisner (2015).

Note finally that the evidence may be inconsistent, or it may be the case that there is no chance function compatible with the evidence which can also be interpreted as a belief function. Which belief functions are compatible with the evidence in such situations? Whilst this question needs answering, it involves some subtleties that are somewhat tangential to the concerns of this paper. For the purposes of this paper, we may simply stipulate that 𝔼 = *ℙ*, the set of all probability functions, in both these cases. See e.g., Williamson (2010, §3.3.1) for a fuller discussion.

### Evidentialism and the Principle of Indifference

Having explicated Evidentialism in terms of BE, we can now move on to its connection to the Principle of Indifference. The important point for our purposes is that BE is inconsistent with the claim that if a belief function is epistemically rational then it satisfies Principle of Indifference PI3. According to BE, any belief function that is compatible with evidence is epistemically rational; there is no further requirement that one’s degrees of belief should equivocate between the basic possibilities. For example, if there is no evidence at all, then any belief function satisfies the evidence, in particular, a belief function *B*_0_ that gives *ω*, one of the basic possibilities, degree of belief 0. BE would deem such a belief function to be epistemically rational. On the other hand, if the claim that epistemic rationality requires PI3 is true then this belief function *B*_0_ is not epistemically rational: one would need to give *ω* degree of belief 1/|Ω| rather than 0. Hence, the claim that epistemic rationality requires PI3 contradicts BE. This claim was the conclusion of the epistemic justification of the Principle of Indifference presented in Section 3. Consequently, the epistemic justification is in tension with Evidentialism.

On the other hand, BE is not inconsistent with the claim that if a belief function is rational *simpliciter* then it satisfies the Principle of Indifference PI3. BE is a claim about *epistemic* rationality, whilst this claim invokes rationality *simpliciter*. This claim is the upshot of the pragmatic justification of the Principle of Indifference of Section 2. Therefore, the pragmatic justification is not in tension with Evidentialism. The advocate of the pragmatic justification can say one of two things about the relation between BE and the claim that rationality requires the Principle of Indifference: either she can point out that there is more to rationality than epistemic rationality, as demonstrated by the cancer example at the start of this section, or she can deny that there is such a thing as ‘epistemic rationality’, perhaps motivated by scepticism about the tenability of a sharp distinction between pragmatic and epistemic rationality or justification. Either way, the proponent of the pragmatic justification of the Principle of Indifference can deny that indifference is a requirement of epistemic rationality. Hence, there is no tension with Evidentialism, under this view.^10^

Before exploring some of the consequences of the conflict between Evidentialism and the epistemic justification of the Principle of Indifference, let us consider a possible response to the claim that there is such a conflict. One might suggest that ‘compatible with the evidence’ should be reinterpreted as follows. Instead of saying that *B* is compatible with the evidence just when *B* ∈ E, one might say that *B* is compatible with the evidence just when *B* = *P* E‡, the probability function in $\mathbb {E}$ with maximum generalised entropy. With this stricter interpretation of ‘compatible with the evidence’, the conflict between Evidentialism and the epistemic justification of Section 3 dissolves, because the epistemic justification implies compatibility with the evidence in the new sense.

The problem with this strategy is that the suggested reinterpretation is not a viable interpretation of ‘compatible with the evidence’. This should already be clear, given the motivation for BE provided above. To highlight how wrong the reinterpretation is, suppose that *E* is correct but is incomplete, i.e., every proposition in *E* is true but *E* does not determine the truth of every proposition. Let *ω*^∗^ be the truth function that represents the true state of the world. Since *E* is correct, *ω*^∗^∈ E. Since *E* is incomplete, there is some proposition *𝜃* such that *ω*^∗^(*𝜃*) = 1 (i.e., *𝜃* is true) but *P* E‡(*𝜃*) < 1.^11^ Under the proposed reinterpretation, *P* E‡ is the only belief function compatible with the evidence. Therefore the belief function that coincides with the truth function *ω*^∗^ is deemed to be incompatible with the evidence. This is perverse: despite the fact that the truth is consistent with the evidence, believing the truth is deemed incompatible with the evidence. The original interpretation of ‘compatible with the evidence’ does not suffer from this problem.

Thus, the proposed reinterpretation is not viable—it strays too far from our usual understanding of ‘compatible’. The conflict between Evidentialism and the epistemic justification of the Principle of Indifference stands.

This epistemic justification is, however, consistent with a significant weakening of the Evidentialism thesis:

**Supervenience.**: One’s beliefs are epistemically rational if and only if they *supervene upon* one’s evidence.

This Supervenience thesis is consistent with the view that compatibility with the evidence is neither sufficient nor necessary for epistemic rationality. Particular implementations of the Supervenience thesis might reinstate necessity, however. One might, for instance, hold that degrees of belief should be compatible with evidence, i.e., *B* ∈ E, and, in addition, *B* should have maximum generalised entropy. In any case, according to Supervenience, the evidence is only a part of the story.

In the literature, it is not always clear whether an author is endorsing the Evidentialism thesis or the Supervenience thesis. Thus it is not always clear whether the inconsistency identified above arises. For example, Mittag (2015, §1) cites Bertrand Russell as an early evidentialist. Russell did indeed say some things which accord with this. For example,

the reason for believing no matter what must be found, after sufficient analysis, in data, and in data alone. Russell (1948, p. 401.)

Despite this, Russell (1948, p. 404) explicitly advocates PI1 and PI2. Since the Principle of Indifference apparently goes well beyond the data, the inconsistency between the epistemic justification of the Principle of Indifference and Evidentialism might seem to pose a problem for Russell. However, it is not entirely clear that Russell was a genuine evidentialist, i.e., an advocate of Evidentialism rather than Supervenience. Without further textual evidence, we cannot conclude that Russell falls foul of this inconsistency.

On the other hand, we have seen that it is indeed clear that a proponent of the epistemic justification of the Principle of Indifference cannot consistently also advocate Evidentialism. We shall suggest next that this inconsistency reflects negatively on the epistemic justification.

## Consequences for consequentialism

Thus far we have seen that the Principle of Indifference can be given a pragmatic justification in terms of avoiding avoidable loss; it can also be given an epistemic justification in terms of reducing inaccuracy; however, this latter form of justification conflicts with Evidentialism. We shall now explore some of the consequences of this tension.

### The pragmatic vs the epistemic interpretation

Let us first turn to the main question of the paper: should the new formal justification of the Principle of Indifference be given a pragmatic reading in terms of loss or an epistemic reading in terms of inaccuracy? I shall argue as follows. Evidentialism is *prima facie* plausible, and, moreover, extant objections to Evidentialism miss the mark. Therefore, the conflict between the epistemic argument and Evidentialism should be taken to reflect negatively on the epistemic argument. On the other hand, the pragmatic version of the justification coheres well with Bayesianism, and as we have seen, is not in tension with Evidentialism. On balance, then, Evidentialism favours the pragmatic interpretation of Section 2 over the epistemic interpretation of Section 3.

First, Evidentialism is, at least *prima facie*, rather plausible. Beliefs can be useful in various ways, as we saw in the cancer example. However, their epistemic value lies in the extent to which they latch on to the truth. In that sense, the epistemic aim of belief is truth. Now, evidence and inference appear to provide our only route to truth. Unless one can infer something about a proposition from one’s evidence, there is no reason to suspect that the proposition is true.^12^ Beliefs are epistemically rational, then, just when they are compatible with (what can be inferred from) evidence.

In order to provide a full defence of Evidentialism, one would need to develop a detailed evidentialist epistemology which validates the principle. This will not be attempted here, for two reasons. First, this is a major enterprise, and not one that can be adequately carried out in the remaining pages of this paper. Second, *any* detailed epistemological theory is likely to involve so many controversial components as to render the whole less plausible than the Evidentialism principle itself. Hence, such a theory will not be very confirmatory.

Instead, I shall aim to explain why key objections related to Evidentialism miss the mark in the sense that they do not help the proponent of the epistemic justification of the Principle of Indifference. Whilst this will not go far enough to establish the truth of Evidentialism, when taken together with the *prima facie* plausibility of Evidentialism it will arguably render Evidentialism in a strong position.

Objections to Evidentialism offer no succour to proponent of the epistemic justification for six main reasons. (i) Some are objections to evidentialist principles other than the Evidentialism thesis as explicated by BE. For example, Sharadin (2016) observes that non-evidential considerations can play a role as motivating reasons for beliefs (a self-fulfilling prophesy, such as belief in survival after cancer, can be such a consideration). However, as Sharadin acknowledges, this fact would not undermine the normative version of Evidentialism set out above (Sharadin 2016, §3). (ii) Some are objections to features of evidentialist epistemologies, rather than to the Evidentialism thesis itself. For example, there are many objections to features and details of Conee and Feldman’s epistemological theory, such as to its internalism (see, e.g., Dougherty 2011, Part V). There are also many objections to Timothy Williamson’s externalist epistemology, such as to its identification of evidence and knowledge (see, e.g., Williamson 2015). These features are not implied by Evidentialism, so problems with these features do not falsify Evidentialism. (iii) Some objections arise from showing that Evidentialism turns out false under one or other non-evidentialist epistemology (see, e.g., Stich 1990; Axtell 2011). As noted above, any detailed epistemological theory will be so controversial as to offer little scope for confirming or undermining Evidentialism. (iv) Some are objections to arguments in favour of Evidentialism, rather than to Evidentialism itself. For example, Steglich-Petersen (2008), Yamada (2010), Sharadin (2016) and Rinard (2017, §8) provide objections to an argument for Evidentialism put forward by Shah (2006). (v) Some are objections which, if successful, would not only undermine Evidentialism but would also undermine Bayesianism, so cannot be used to favour the epistemic argument over Evidentialism for the purposes of justifying the Principle of Indifference. For example, as discussed in Section 4, DeRose (2000, §1) and Baehr (2009) are concerned that Evidentialism handles too weak a sense of epistemic rationality. However, the weak sense is precisely the sense of epistemic rationality that Bayesianism tackles. (vi) Some objections, if successful, would not only undermine Evidentialism but would also undermine the epistemic argument so do not help proponents of such a justification of the Principle of Indifference. For example, Marušić (2012) argues that decisions and promises require believing propositions which may be contrary to the evidence. If so, these are beliefs which are also more inaccurate than those in accord with the evidence. To take another example, Littlejohn (2012, §7.3) argues against Evidentialism by claiming that belief is factive. As we shall see below (c.f. principle I5), this claim is as unattractive to the proponent of an inaccuracy argument as it is to the evidentialist.

We have seen that objections to Evidentialism fail to help the proponent of the epistemic justification of the Principle of Indifference. Since Evidentialism is *prima facie* plausible, the fact that Evidentialism is in tension with the epistemic justification casts some doubt on that justification.

On the other hand, no such doubt is cast on the pragmatic version of the justification of PI3. This is because, as we saw above, there is no tension between Evidentialism and the pragmatic justification. Furthermore, the pragmatic justification is relatively unproblematic because it rests on the idea that one should avoid avoidable loss, and this goal is central to Bayesianism. The main existing argument for Probabilism—the Dutch book argument (de Finetti 1937)—is based on exactly this premiss, since it shows that degrees of belief must be probabilities if one is avoid avoidable sure loss in a particular betting set-up. Moreover, Bayesianism is intended as a practical theory which can guide decision making, and the supposition behind Bayesian decision theory is that one should avoid avoidable loss by maximising expected utility. The pragmatic reading of the justification of the Principle of Indifference is clearly also of the form *avoid avoidable loss*: in this case, *avoid avoidable worst-case expected loss*. Hence, the pragmatic reading coheres well with Bayesianism.

In sum, then, the epistemic justification of Section 3 comes out worse than the pragmatic justification of Section 2 from its clash with Evidentialism.

### Other epistemic justifications of the Principle of Indifference

It is worth noting that the epistemic argument of Section 3 is not the only epistemic argument for the Principle of Indifference. Pettigrew (2016b), for example, gives another argument in terms of epistemic inaccuracy. This argument is for PI1; recall that this says that when there is no evidence one should believe each basic possibility to the same extent. Suppose, then, that there is no evidence, *E* = *∅*. Pettigrew supposes that a measure *I*(*ω*,*B*) of inaccuracy measure should satisfy the following two requirements. First, there is no other belief function *B* that has as low inaccuracy as the equivocator function for all basic possibilities: if *B*≠*P*_=_ then there is some *ω* such that *I*(*ω*,*B*) > *I*(*ω*,*P*_=_). Second, Pettigrew requires that *I* is invariant under isomorphisms acting on the set of propositions. (As with the earlier requirement that a weighting function be unbiased, this second requirement ensures that inaccuracy measures are invariant under permutations of the basic possibilities.) The first requirement forces the equivocator function to be less inaccurate than *B* for some basic possibility, and the second forces the inaccuracy of the equivocator function to be the same for every basic possibility. Hence the equivocator function is the belief function with minimum worst-case inaccuracy, where the worst case is taken over all *ω*. Thus, Pettigrew argues, PI1 must hold if one is to minimise worst-case inaccuracy.

It is important to observe that the grounds for preferring the pragmatic justification of Section 2 over the epistemic version of Section 3 also apply to other epistemic justifications of the Principle of Indifference, such as Pettigrew’s justification. This is because it is the claim that epistemic rationality requires the Principle of Indifference that is inconsistent with Evidentialism, and any epistemic justification of the Principle of Indifference will yield that claim. Thus, if this inconsistency favours the pragmatic justification of Section 2 over the epistemic justification of Section 3, it also favours the pragmatic justification of Section 2 over any other epistemic justification of the Principle of Indifference.

A further worry is relevant here. Any other epistemic justification of the Principle of Indifference that appeals to inaccuracy will need to provide an account of inaccuracy that is somewhat different to that given in Section 3. Such an account of inaccuracy will hinge on a complex package of claims which can be hard to justify. This complexity, when contrasted with the simplicity and prima facie plausibility of Evidentialism, may cast further doubt on the inaccuracy account.

To get a sense of this complexity, consider that any account of epistemic inaccuracy which underpins a purely epistemic justification of the Principle of Indifference will depend upon the following claims I1–5, which I shall collectively call the *Inaccuracy Package*:

- **I1**: Some specific function *f* can measure the inaccuracy of a belief function.
  In the case of our epistemic justification of Section 3, a logarithmic function was singled out as most appropriate. Pettigrew (2016b) favours the quadratic Brier score, though his justification of PI1 considered a class of inaccuracy measures. Different classes of inaccuracy measures have appeared in the literature, often delineated by technical fruitfulness rather than philosophical considerations—e.g., ‘strictly proper’ inaccuracy measures are particularly conducive to proving the required theorems (Landes 2015). As yet, we are far from a consensus as to which functions are appropriate as inaccuracy measures. Worse, it is controversial whether inaccuracy is the sort of thing which can be measured by a single number, and which depends only on the belief function in question and the true state of the world. It is still an open question whether the quest for an inaccuracy measure will turn out to be as quixotic as the closely related quest for a measure of verisimilitude. This stands in contrast to loss (negative utility), which is so well entrenched within the Bayesian framework that Bayesians find it uncontroversial that one can measure loss by a single number that depends on the belief function and the state of the world.
- **I2**: Some unique norm *N*(*f*) governs inaccuracy.
  In the epistemic justification of Section 3, the norm is to minimise worst-case expected *f*. In Pettigrew’s justification, it is to minimise worst-case *f*. Another norm often invoked by proponents of inaccuracy arguments is to avoid dominated *f*. Note that these norms conflict; we saw that the first norm warrants PI3 but the second norm does not. Apparently, the third norm, which can be used to motivate Probabilism, cannot be used to justify even PI1 (Pettigrew 2016a, Chapter 12). Therefore, the proponent of inaccuracy arguments needs to provide grounds for singling out which norm, or which combination of norms, should be applied. This has not been done as yet.
- **I3**: *N*(*f*) is a purely epistemic standard.
  Inaccuracy needs to be a purely epistemic standard if it is to provide a purely epistemic justification of the Principle of Indifference. Now, the word ‘inaccuracy’ has epistemic connotations, but in the light of the epistemic justification of Section 3, which characterises the inaccuracy measure in just the same way that the measure of loss was characterised in Section 2, one might worry whether inaccuracy is just loss in disguise. This casts some doubt on whether inaccuracy is a purely *epistemic* consideration. Is minimisation of inaccuracy an appropriate *standard*? Truth is uncontroversially an epistemic standard, but a small improvement in the accuracy of a belief is less obviously so—in many circumstances, a miss is as good as a mile, and one might hold that truth is the only epistemic standard to which beliefs should conform.
- **I4**: *N*(*f*) is necessary for rationality of belief.
  Truth is an epistemic standard but it is not normally thought of as necessary for rationality of belief. Even if minimisation of inaccuracy is an epistemic standard, some further consideration—currently lacking—needs to be provided before we can be convinced of its necessity for rationality. Furthermore this consideration needs to be clearly epistemic. Suppose our beliefs fit our evidence; should we follow norm *N*(*f*) in addition? *f* doesn’t tell us any more about what is true than the evidence does. Of course, we might worry that we’ll lose out in proportion to our inaccuracy—but then avoiding inaccuracy is a pragmatic desideratum, rather than an epistemic one.
- **I5**: *N*(*f*) is sufficient for rationality of belief.
  Now, *N*(*f*) can only be sufficient for epistemic rationality if no other norm is necessary. One concern for sufficiency is a slippery slope: if accuracy is necessary for rationality, then why isn’t truth also necessary? The proponent of inaccuracy arguments needs to say why such a move is not warranted. The proponent will want to resist such a move, not only because requiring truth over and above controlling inaccuracy contradicts the sufficiency of *N*(*f*), but also because if rational belief is factive then very implausible principles of indifference will follow. For example, suppose that truth were also necessary for rationality of belief in the sense that a degree of belief in basic possibility *ω* which surpasses some threshold *τ*, *P*(*ω*) > *τ*, is epistemically rational only when *ω* is true. Unless the evidence *forces* the truth of some possibility *ω*, it is impossible to determine for sure whether *ω* is true, and hence whether it is epistemically rational to set *P*(*ω*) > *τ*. Hence, the Bayesian can normally only be sure of following this truth norm by setting *P*(*ω*) ≤ *τ* for every *ω*, i.e., by being sufficiently indifferent between the basic possibilities. In cases where, for some *ω*, evidence fails to force the truth of *ω* but implies that the chance of *ω* exceeds the threshold, *P*^∗^(*ω*) ≥ *τ*, this truth norm violates the requirement that *P* ∈ E. Advocating degrees of belief which are incompatible with the evidence clearly goes much further than most Bayesians would like.

There is another, more well known, worry about I4–5, presented by Greaves (2013, p. 918):

Emily is taking awalk through the Garden of Epistemic Imps. Achild plays on the grass in front of her. In anearby summerhouse are *n* further children, each of whom may or may not come out to play in aminute. They are able to read Emily’s mind, and their algorithm for deciding whether to play outdoors is as follows. If she forms degree of belief 0that there is now achild before her, they will come out to play. If she forms degree of belief 1that there is achild before her, they will roll afair die, and come out to play iff the outcome is an even number. More generally, the summerhouse children will play with chance (1 − 12*q*(*C*_0_)), where *q*(*C*_0_) is the degree of belief Emily adopts in the proposition (*C*_0_) that there is now achild before her. Emily’s epistemic decision is the choice of credences in the proposition *C*_0_ that there is now achild before her, and, for each *j* = 1,…,*n*, the proposition *C*_*j*_ that the *j* th summerhouse child will be outdoors in afew minutes’ time.

The problem is as follows. Assume Emily knows all the facts set out above. Emily’s evidence determines that *C*_0_ is true. The Bayesian will want to say that she should fully believe *C*_0_. Then, for *j* = 1,…,*n*, the chance of each *C*_*j*_ will be 12 and the Bayesian will prescribe degree of belief 12 in each *C*_*j*_. These latter degrees of belief will be deemed inaccurate by some typical inaccuracy measures *f*, leading to low overall accuracy. Much greater total accuracy would be achieved if Emily were to fully disbelieve *C*_0_, contrary to her evidence, and fully believe *C*_1_,…,*C*_10_, which would then all be true. Now, if following *N*(*f*) were necessary and sufficient for rationality of belief then Emily should adopt the latter beliefs. Such a norm, which requires violating the evidence, would be very unpalatable to the Bayesian.^13^

Such examples strengthen the conflict between Evidentialism and inaccuracy arguments. Evidentialism states that compatibility with the evidence is necessary and sufficient for rationality of beliefs. Inaccuracy arguments can conflict both the necessity and sufficiency of this claim: whilst the fact that inaccuracy arguments are used to justify the Principle of Indifference contradicts sufficiency, the Epistemic Imps example tells against necessity.

The Inaccuracy Package, then, is not only complex, it is also riddled with lacunae and challenges. This is not to say that all these challenges are insuperable, rather that, currently, the Inaccuracy Package is merely a promissory note. On the other hand, I have argued that Evidentialism is *prima facie* plausible and objections to Evidentialism miss the mark. Consequently, Evidentialism is more credible than the Inaccuracy Package, and, because of the inconsistency between inaccuracy arguments and Evidentialism, a pragmatic justification of the Principle of Indifference will be on firmer ground than any epistemic justification which appeals to inaccuracy.

Let us consider a response to this line of argument, to be found in some comments of Pettigrew (2016b, §3.1). Pettigrew expresses the worry that evidentialists need to invoke multiple cognitive goals. For example, it is not enough to fit our beliefs to the evidence we have—we ought to gather new evidence too. On the other hand, proponents of inaccuracy arguments need only invoke a single cognitive virtue, namely accuracy. According to Pettigrew, the single goal of accuracy explains both the need to fit evidence and the need to gather new evidence because they both help to reduce inaccuracy. Thus the Inaccuracy Package should be preferred over Evidentialism on the grounds that the former is more explanatory than the latter.

There are various compelling rejoinders open to the evidentialist, however. First, avoiding inaccuracy fails to explain the need to fit evidence. As Pettigrew acknowledges, examples like the Epistemic Imps example set out above show that one should not always fit the evidence if one is to minimise inaccuracy. Second, avoiding inaccuracy also fails to explain the need to gather new evidence. If accuracy were the only goal, one should not gather new evidence wherever that evidence is likely to lead to less accurate degrees of belief—i.e., one should not gather evidence that is likely to reveal that the chances are such as to lead to higher values of the chosen inaccuracy measure *f* when degrees of belief are calibrated to those chances. Third, although the proponent of inaccuracy arguments might claim that the inaccuracy approach is more explanatory, that plays into the hands of the evidentialist, whose objection is precisely that inaccuracy arguments *explain too much*: they explain pragmatic norms such as the Principle of Indifference when they should only be explaining epistemic norms. Fourth, it is far from clear that the evidentialist fails to explain both the need to gather evidence and the need to fit beliefs to evidence. BE cashes out Evidentialism in terms of calibration of degrees of belief to chances. Better calibration to chances can explain the need to gather more evidence of chances as well as to fit degrees of belief to current evidence of chances.

Consequently, Pettigrew’s response does not succeed. The inconsistency between Evidentialism and inaccuracy arguments for the Principle of Indifference does indeed favour the former over the latter. A Bayesian seeking a justification for the Principle of Indifference should prefer a pragmatic justification over the epistemic justification of Section 3.

### Inaccuracy arguments in general.

We have seen that inaccuracy arguments for the Principle of Indifference are problematic, and that pragmatic arguments arguably fare better in motivating the Principle of Indifference. But these worries extend beyond the Principle of Indifference. This is because inaccuracy arguments for the Principle of Indifference are of exactly the same kind as inaccuracy arguments for other norms of Bayesianism—not only Probabilism but also norms, such as the Principal Principle, which require calibration of degrees of belief to chances (see, e.g., Joyce 1998; Leitgeb and Pettigrew 2010a, b). If these arguments fail with respect to the Principle of Indifference, then that is a problem for the Inaccuracy Package in general. The whole project of using inaccuracy arguments to provide an epistemic consequentialist justification of Bayesianism is brought into question.

One possible response to this problem is to somehow differentiate inaccuracy arguments for the Principle of Indifference from those for other Bayesian norms, in order to prevent objections to the former from affecting the latter. Perhaps the most promising point of differentiation is in the choice of norm—I2 of the Inaccuracy Package. If one restricts inaccuracy arguments by appealing solely to avoiding dominance of inaccuracy, not worst-case inaccuracy nor worst-case expected inaccuracy, one may hope to firewall Probabilism and the Principal Principle from the inconsistency with Evidentialism. As noted above, avoiding dominance of inaccuracy can be used to justify Probabilism and the Principal Principle, but not the Principle of Indifference. The proponent of inaccuracy might then accept that the Principle of Indifference is a pragmatic norm, but maintain that Probabilism and the Principal Principle are epistemic norms, to be justified in terms of avoiding inaccuracy. But what are the grounds for dismissing the suggestion that one should minimise worst-case inaccuracy or minimise worst-case expected inaccuracy? As yet, we are lacking a principled answer to this question.

Even if a principled response can be given to this question, proponents of inaccuracy arguments are left with a further question: why should some norms of Bayesianism—e.g., Probabilism—be given a non-pragmatic justification and others—such as the Principle of Indifference—a pragmatic justification? Having two forms of justification seems otiose, especially when one can get away with a single, unified, pragmatic justification of all the norms of Bayesianism, such as that provided in Section 2.

### Epistemic Consequentialism

The conflict between Evidentialism and inaccuracy arguments for Bayesianism renders the latter implausible, if the above reasoning is sound. But inaccuracy arguments for Bayesianism are the best available epistemic consequentialist arguments for Bayesianism. So epistemic consequentialism in general is on thin ice. If Bayesianism can’t be justified in terms of its epistemic consequences, the pragmatic approach remains the most promising.

Of course epistemic consequentialism isn’t a one-horse race. Reliabilism is an alternative option to inaccuracy arguments for Bayesian norms. In the context of degrees of belief, the reliabilist approach has been much less thoroughly investigated than the inaccuracy approach, but Dunn (2015) has suggested that the reliabilist approach is preferable to the inaccuracy approach. Besides taking issue with the inaccuracy approach, Dunn argues for an alternative that measures the reliability of a belief-formation process by how well calibrated the resulting degrees of belief are to chances. There are three main worries about this sort of approach, as we shall now see.

First, a Bayesian calibration norm is one of the things that we would like to justify—a justification which itself appeals to calibration is hardly likely to be very convincing there.

Second, it appears that this reliability account might conflict with Probabilism, which would be a serious concern for the Bayesian.^14^ Suppose that a visual process is correct 95% of the time and an auditory process is correct 80% of the time. Someone who sees no evil and hears no evil would be perfectly rational, under this sort of reliabilist approach, to believe that there is no evil to degree 0.95 *and* to degree 0.8—i.e., to believe the same proposition to two different degrees, contradicting Probabilism. One might try to save Probabilism by modifying the account to characterise rationality in terms of the reliability of the process determining the belief function as a whole, rather than individual degrees of belief. In our example, if the prior probability that there is no evil present is 0.5, then its posterior probability, given that evil is neither seen nor heard, is about 0.99, so this latter (unique) degree of belief would be rational.^15^ But then Probabilism follows too easily—it follows directly from the stipulation that the one should focus on the reliability of the belief function as a whole, together with the stipulation that rationality is assessed in terms of calibration to chances and the assumption that chances are probabilistic. For such a move to be convincing, the two stipulations would need to be given some independent motivation. This is currently lacking.

The third worry about this reliabilist approach is that, although it is unclear whether or not such a line of argument will extend to justifying the Principle of Indifference, difficulties arise either way. On the one hand, so-called ‘concentration theorems’ suggest that probability functions that are indifferent (i.e., have maximum entropy) are overwhelmingly likely (see, e.g., Jaynes 2003, §11.4), so perhaps a case can be made for an indifferent belief function being better calibrated to chances. But then there would be a conflict between reliabilism, which would deem the Principle of Indifference to be an epistemic norm, and Evidentialism, which would class it as non-epistemic. This takes the epistemic consequentialist back to square one, a conflict with Evidentialism. On the other hand, if the Principle of Indifference does not admit a reliabilist justification, the epistemic consequentialist is left with the problem of motivating a mixture of pragmatic and epistemic justifications for different Bayesian norms, instead of simply adopting a unified pragmatic justification, such as that of Section 2.

Given the current state of play, then, Bayesianism is best motivated pragmatically. Epistemic consequentialism remains an interesting project, but there is a lot more to do before either an approach based on the Inaccuracy Package or a reliabilist approach can offer a viable alternative to pragmatic justifications of Bayesian norms in terms of avoiding avoidable losses.

## Footnotes

1 A set of probability functions is convex if, for any two probability functions *P* and *Q* that are in the set, any convex combination *R* of *P* and *Q*, defined by *R*(*ω*) = *λ**P*(*ω*) + (1 − *λ*)*Q*(*ω*) for some *λ* ∈ [0, 1] and each *ω* ∈Ω, is also in the set. Non-convex sets introduce complexities which would obscure the main points of this paper, and as we shall see later, they do not affect the key results.

2 *E* is *invariant under permutations of the**ω* ∈Ω if for any permutation *π* on Ω, permuting each *ω* to *π*(*ω*) in each statement in *E* yields the same set $\mathbb {E}$ of probability functions. This corresponds to rotational symmetry of 𝔼 in the simplex $\mathbb P$: symmetry under rotation by 120^∘^ in Fig. 2.

3 To see this, suppose that *E* is invariant under permutations of Ω. Because $\mathbb {E}$ is non-empty, convex and invariant under rotational symmetry, *P*_=_ ∈ E. Hence by PI3, *P* = *P*_=_, as required. Note that PI3 only implies PI2 under the convexity assumption. Without convexity, PI2 is implausible, because in that case PI2 may require that *P*∉E, i.e., that *P* should lie outside the set 𝔼 of probability functions that are compatible with the evidence.

4 We thus presume that the chance function *P*^∗^ is a probability function. The definition above presupposes that the chance function is defined on the same domain Ω as the agent’s belief function *P*. However, this presupposition is not essential. Suppose instead that the chance function is defined on some other domain, which may include certain propositions not expressible in Ω and which may not include certain propositions expressible in Ω. In that case, we take the set evidentially-compatible chance functions to be the set 𝔼 of probability functions on domain Ω that are *consistent* with evidence of chances in *E*: any *Q* ∈ 𝔼 is consistent with evidence of chances in *E* and any probability function *Q* defined on Ω that is consistent with evidence of chances in *E* is in the set E. As noted in the previous section, we do not assume that the evidence *E* consists of propositions expressible in Ω.

5 The quantity *L*(*F*,*B*) is not interpreted as a formal expectation, for two reasons. First, since one of our goals is to motivate Probabilism, we are not in a position to presuppose that any such belief distribution is a probability function, which it would need to be in order to define a formal expectation. To get round this problem, one might suggest that one could generalise the notion of formal expectation to weight the loss by normalised degree of belief, rather than probability. However, a second problem would then arise, as follows. The expectation would need to be an average, taken over all possible decision scenarios, of the loss incurred in each scenario, weighted by the degree to which one believes that scenario will obtain. It is unrealistic to think that one could enumerate all possible decision scenarios (i.e., all possible losses as well as all possible sets of options) and specify a degree of belief in each scenario obtaining. Certainly, we shall not assume here that the domain of the agent’s belief function is large enough to include every possible decision scenario.

6 Here we take the expectation with respect to chance, *P*^∗^, rather than normalised degree of belief, *B*. Partly, this is because chance is assumed to be probabilistic but *B* is not. This is in line with the standard view of scoring rules in the statistical literature (e.g., Grünwald and Dawid 2004), though some works in the philosophical literature do weight by non-probabilistic belief (e.g., Leitgeb and Pettigrew 2010a). More fundamentally, we focus on the concept of objective expectation rather than subjective expectation on the grounds that: (i) objectively expected loss gives a better estimate of actual loss than subjectively expected loss where the two disagree; and (ii) evidence tells us something about the chances (namely that *P*^∗^∈ E) so objectively expected loss is sufficiently accessible for our purposes. One might counter that—just as objectively expected loss is preferable to subjectively expected loss—it would equally be better to focus on the actual loss function rather than anticipated loss *L*(*F*,*B*) = −*k* log *B*(*F*). However, the actual loss function is not normally known, even to within some well circumscribed subset of possible loss functions. The fact is that whilst evidence usually tells us quite a lot about chances, it rarely tells us much about the losses we will be incurred in all future decision scenarios.

7 Each *F* ⊆Ω is in precisely one two-membered partition, namely {*F*,*F̄*}, so

$$S_{{\mathcal P}{\Omega}} (P^{*},B) = \sum\limits_{F\subseteq {\Omega}} P^{*}(F) L(F,B) = \sum\limits_{\pi =\{F, \bar{F}\}} 1 \sum\limits_{G\in \pi} P^{*}(G) L(G,B).$$

8 This follows because *P*^∗^(*s*) = *P*^∗^(*s*|*g*)*P*^∗^(*g*) + *P*^∗^(*s*|¬*g*)*P*^∗^(¬*g*) = 0.8 × 0.7 + *P*^∗^(*s*|¬*g*) × 0.3 and *P*^∗^(*s*|¬*g*) ∈ [0, 1].

9 Other pragmatic considerations may apply to the doctor who is treating the patient. If the arguments of Section 2 are sound then one might argue that the doctor ought to believe *s* to some degree sufficiently close to 0.71, the maximum entropy value. Again, this is for pragmatic reasons—other values incur an avoidable increase in worst-case expected loss.

10 On the second view—denying epistemic rationality—the proponent of a pragmatic justification of the Principle of Indifference will deny the force of Evidentialism as well as the force of the epistemic justification.

11 The probability function with maximum generalised entropy will give a proposition that is not constrained to have probability 1 a probability lower than 1—see Landes and Williamson (2013, Proposition 18).

12 Perhaps certain logical or mathematical truths can be inferred without evidence. But then they can trivially also be inferred from evidence. Perhaps certain other propositions are self-evident. But then they too can be inferred from evidence.

13 Easwaran and Fitelson (2012) present some other situations in which avoiding inaccuracy requires violating the evidence.

14 I am very grateful to Jeff Dunn for pointing this out.

15 Let *e*, *s* and *h* denote respectively that there is no evil present, no evil has been seen, no evil has been heard. Suppose that seeing and hearing evil are probabilistically independent conditional on the presence or absence of evil. We have that *P*(*e*) = 0.5,*P*(*s*|*e*) = *P*(¬*s*|¬*e*) = 0.95,*P*(*h*|*e*) = *P*(¬*h*|¬*e*) = 0.8. Hence,

$$P(e|sh)\! =\! \frac{P(s|e)P(h|e)P(e)}{ P(s|e)P(h|e)P(e) \,+\, P(s| \neg e)P(h| \neg e)P(\neg e)} \,=\, \frac{0.95\!\times\! 0.8\times 0.5}{0.95\!\times\! 0.8\!\times\! 0.5 \,+\, 0.05\times 0.2\times 0.5} \approx 0.987.$$
